# Safety and reactogenicity of a controlled human infection model of sand fly-transmitted cutaneous leishmaniasis

**DOI:** 10.1038/s41591-024-03146-9

**Published:** 2024-08-02

**Authors:** Vivak Parkash, Helen Ashwin, Shoumit Dey, Jovana Sadlova, Barbora Vojtkova, Katrien Van Bocxlaer, Rebecca Wiggins, David Thompson, Nidhi Sharma Dey, Charles L. Jaffe, Eli Schwartz, Petr Volf, Charles J. N. Lacey, Alison M. Layton, Paul M. Kaye

**Affiliations:** 1 York Biomedical Research Institute, Hull York Medical School, University of York, York, UK; 2 Department of Parasitology, Faculty of Science, Charles University, Prague, Czech Republic; 3 Skin Research Centre, Hull York Medical School, York, UK; 4 York and Scarborough Teaching Hospitals NHS Foundation Trust, York, UK; 5 Department of Microbiology and Molecular Genetics, Kuvin Center for the Study of Infectious and Tropical Diseases, IMRIC, The Hebrew University – Hadassah Medical School, Jerusalem, Israel; 6 Center for Geographic Medicine and Tropical Diseases, Chaim Sheba Medical Center and the School of Medicine, Tel Aviv University, Tel Aviv, Israel

**Keywords:** Parasitic infection, Vaccines

## Abstract

The leishmaniases are globally important parasitic diseases for which no human vaccines are currently available. To facilitate vaccine development, we conducted an open-label observational study to establish a controlled human infection model (CHIM) of sand fly-transmitted cutaneous leishmaniasis (CL) caused by *Leishmania major*. Between 24 January and 12 August 2022, we exposed 14 participants to *L. major*-infected *Phlebotomus duboscqi*. The primary objective was to demonstrate effectiveness of lesion development (take rate) and safety (absence of CL lesion at 12 months). Secondary and exploratory objectives included rate of lesion development, parasite load and analysis of local immune responses by immunohistology and spatial transcriptomics. Lesion development was terminated by therapeutic biopsy (between days 14 and 42 after bite) in ten participants with clinically compatible lesions, one of which was not confirmed by parasite detection. We estimated an overall take rate for CL development of 64% (9/14). Two of ten participants had one and one of ten participants had two lesion recurrences 4–8 months after biopsy that were treated successfully with cryotherapy. No severe or serious adverse events were recorded, but as expected, scarring due to a combination of CL and the biopsy procedure was evident. All participants were lesion free at >12-month follow-up. We provide the first comprehensive map of immune cell distribution and cytokine/chemokine expression in human CL lesions, revealing discrete immune niches. This CHIM offers opportunities for vaccine candidate selection based on human efficacy data and for a greater understanding of immune-mediated pathology. ClinicalTrials.gov identifier: NCT04512742.

## Abstract (web summary)

The first controlled human infection model of cutaneous leishmaniasis transmitted by sand flies enables assessment of immune responses and parasitism in infected participants that can inform future vaccine and therapeutic development.

## Main

The leishmaniases are vector-borne diseases transmitted by phlebotomine sand flies^1^, with a global impact on health and well-being^2^. Several species of *Leishmania* infect humans, causing a spectrum of tegumentary and systemic diseases^1^. Cutaneous leishmaniasis (CL) is endemic in 89 countries reporting to the World Health Organization (WHO), and over 200,000 new autochthonous cases were reported in 2020, which is widely regarded as a substantial underestimate^3^. CL presents as an inflammatory lesion at the site of transmission, but clinical outcome is typically dependent on parasite species. Lesions due to *L. major* commonly self-resolve over several months and/or respond well to topical therapy. The resulting scar may, however, have lifelong impact on well-being^4–6^. Lesions due to *Leishmania* *tropica* and *Leishmania* *mexicana* are more chronic, persist often for years and can be refractory to treatment. Some species have metastatic potential (for example, *Leishmania* *braziliensis* and *Leishmania* *guyanensis*), causing mucocutaneous leishmaniasis, whereas others spread within the skin, causing disseminated or diffuse leishmaniasis (for example, *Leishmania* *aethiopica*). *Leishmania* *donovani* and *Leishmania* *infantum* typically disseminate systemically, causing life-threatening visceral leishmaniasis (VL). With VL declining in South Asia, East Africa now carries the major burden of VL. Case fatality rates (2–3%) have also changed little over the past decade, and poorer outcomes are reported in some countries^3^. Because vector control alone is likely to be insufficient, controlling the leishmaniases by vaccination is an important goal.

The potential for a vaccine to reduce the public health burden of leishmaniasis is well recognized^7,8^. A recent WHO report identified *Leishmania* as the highest priority parasitic target for new vaccine development after *Plasmodium falciparum* malaria^9^, and vaccine development is supported by recent estimates of the global demand for^10^ and affordability of^11^ a successful leishmaniasis vaccine. However, despite decades of effort and numerous animal studies^12,13^, few *Leishmania* vaccine candidates have progressed to clinical trial^8^. Only two are currently in clinical development: an adenovirus-vectored vaccine encoding two *Leishmania* antigens (ChAd63-KH^14^) and a live genetically attenuated vaccine (*L. major* cen^−/−^ (ref. ^15^)). Many factors adversely impact vaccine development^12^; thus, new approaches are needed to identify and validate candidate vaccines, improve understanding of natural and vaccine-induced protection in humans and ultimately shorten the pathway to registration. Controlled human infection models (CHIMs, also known as controlled human infection studies) can address these issues directly.

CHIMs are now well embedded in the vaccine development pathway, including for malaria^16,17^, schistosomiasis^18^ and hookworm^19^. Deliberate human infection with *Leishmania* is not new^20^. Leishmanization, the inoculation of lesion scapings into cosmetically hidden areas, has been practiced for centuries in CL-endemic countries to induce immunity and minimize visible scarring and stigma^21^. Leishmanization using needle challenge was evaluated for vaccine development in the early 2000s^22^ but was not pursued. Subsequently, the importance of vector-associated immune modulation has become appreciated^23^, with evidence suggesting that vaccines effective against needle challenge may not protect against natural transmission^24^.

Here we report a CL CHIM that incorporates natural sand fly transmission. The study primary objectives were to determine the proportion of exposed individuals who developed CL lesions (take rate) and safety (absence of lesions at 12-month follow-up). We report that this model was effective, safe and well tolerated by study participants. In addition, analysis of lesion biopsies provides new insights into the immune landscape associated with early CL in humans.

## Results

### Study design

**Fig. 1:**
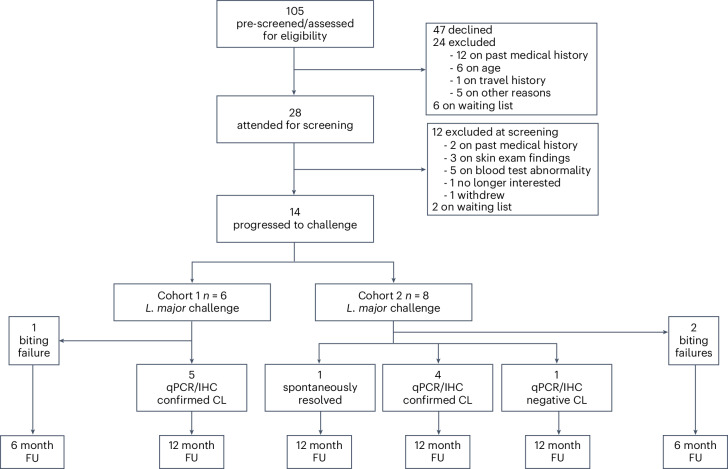
CONSORT diagram summarizing the LEISH_Challenge study. A CONSORT checklist is included in Supplementary Information. Details of participant demographics are provied in Supplementary Table 1. Study protocol and the participant information sheet are provied in Supplementary Information. FU, follow-up.

We previously reported on enabling studies, including development of a cGMP challenge strain (*L. major* MHOM/IL/2019/MRC-02)^25^, development of a sand fly biting protocol^26^ and assessments of public perceptions of the project^27^. For this first human infection study (LEISH_Challenge; ClinicalTrials.gov identifier: NCT04512742), between 23 November 2021 and 4 August 2022, we enrolled 14 healthy *Leishmania*-naive volunteers aged 18–50 years at the University of York Translational Research Facility (Fig. 1 and Supplementary Table 1). The first and last participants were exposed to infected sand flies on 24 January and 12 August 2022, respectively. There were eight female and six male participants, median 32 years old, all White ethnicity, reflecting our local resident population (98% White ethnicity). Sex was not explicitly factored into the experimental design. Sex was assigned by researchers, and gender was not recorded. Exclusion and inclusion criteria were as per study protocol (Supplementary Information) and included absence of *Leishmania* exposure history, willingness to refrain from travel to *L. major*-endemic regions during the study and absence of significant atopy or active skin disease. All participants had negative HIV, hepatitis B and hepatitis C serology and gave written informed consent. A pragmatic adaptive design was chosen to expose the least number of participants to infection and provide flexibility based on initial outcomes. Five *Phlebotomus duboscqi* sand flies infected with *L. major* were allowed access for 30 min to the volar aspect of the proximal forearm, approximately 2–3 cm distal to the antecubital fossa, using a bespoke biting chamber with a variable aperture^26^. Participants were considered for therapeutic biopsy when a clinically apparent lesion of ≥3-mm diameter was observed. However, biopsy could not always be performed on the day of evaluation and was often postponed until another study visit could be scheduled. All participants were followed up for 12 months and thereafter advised to contact the study team and their general practitioner in case of future recurrence.

### Development of CL after sand fly exposure

**Fig. 2:**
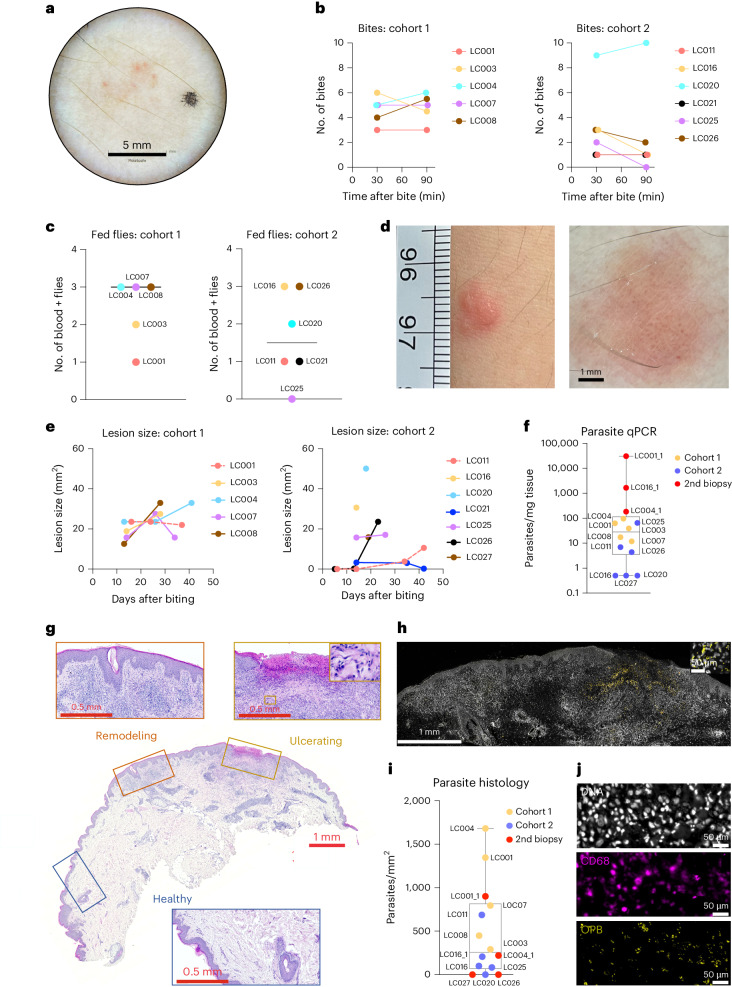
Parasitological outcomes in the LEISH_Challenge study. **a**, Representative dermoscopy image showing bite sites. **b**, Number of recorded bites per participant per cohort at 30 min and 90 min. No significant differences were noted between cohorts at either 30 min (*P* = 0.104) or 90 min (*P* = 0.08) (two-sided Mann–Whitney test). *n* = 5 (cohort 1) and *n* = 6 (cohort 2). **c**, Number of partially and/or fully blood-fed sand flies after biting per participant per cohort (*P* = 0.36; two-sided Mann–Whitney test). *n* = 5 (cohort 1) and *n* = 6 (cohort 2). Bar represents the median. **d**, CL lesion development and associated dermoscopy image (LC004, 13 d p.b.). **e**, Lesion areas (mm^2^) at varying times after bite per participant per cohort. **f**, Parasite load per milligram of biopsy tissue. Individual symbols reflect a single participant/biopsy (*n* = 14); box and whisker plot with median and maximum/minimum values. **g**, H&E-stained biopsy tissue from LC001, highlighting histologically normal tissue, a potential bite site with epidermal remodeling and an ulcer. Box in the upper right image indicates higher magnification view of area of parasitism. Representative H&E-stained sections from all other participants are shown in Extended Data Fig. 1. Scale bar, as indicated. At least two independent sections from each participant were studied. **h**, IHC for *Leishmania* OPB (yellow) counterstained for nuclei (YOYO-1; white). Area shown represents remodeling and ulcerated regions from serial section to that shown in **g**. Box shows higher magnification of area of parasitism. **i**, Parasites per mm^2^ of tissue was determined by quantitative morphometry. Symbols show each participant and respective cohort (*n* = 14); box and whisker plot with median and maximum/minimum values. **j**, IHC for OPB (yellow) and CD68 (purple) to show intracellular parasitism. Parasites are also evident by nuclear staining (YOYO1; white) with characteristic nucleus/kinetoplast. Scale bar, 50 μm. p.b., post-bite.

**Extended Data Fig. 1:**
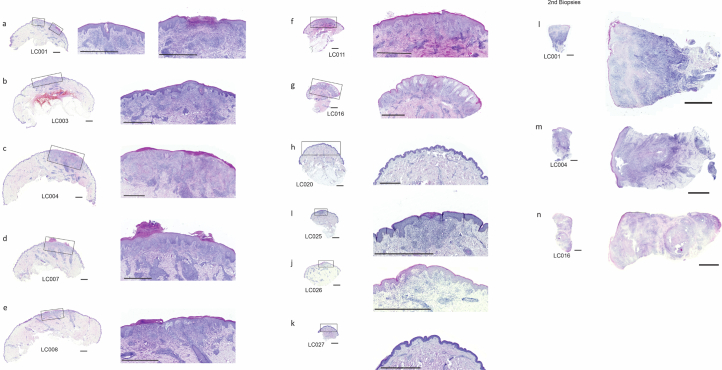
H&E-stained FFPE sections from each biopsy. **a-n**. H&E stained FFPE sections from all biopsied participants. A single section is shown per participant, representative of at least three examined. Boxed areas are shown at higher magnification. All scale bars represent 1mm.

For the first six participants, we used a biting chamber aperture of 6-mm diameter. One of six participants (17%; LC012) was deemed a bite failure (Methods) but developed a small 2-mm-diameter papule at the exposure site 4 weeks later. This persisted for 3 weeks and was removed by punch biopsy but showed no evidence of CL or clinically significant histological abnormality. The remaining five of six participants had 1–6 confirmed bites (Fig. 2a,b) received from 1–3 sand flies (Fig. 2c), and all developed a lesion with clinical appearance of CL (median area at days 13–16 of 18.9 mm^2^; range, 12.6–23.6 mm^2^; Fig. 2d,e). Dermoscopy supported a diagnosis of leishmaniasis, showing characteristic erythema, teardrop‑like structures, hyperkeratosis and vascular structures, which included linear, dotted and hairpin‑like vessels^28^. Therapeutic excision biopsy was performed between day 28 and day 41 after bite (median, 34 d) allowing for confirmation of CL by quantitative polymerase chain reaction (qPCR) and/or immunohistochemistry (IHC). Five of five biopsies were positive by qPCR, with a high degree of variance in parasite load (median, 1,218 parasites per milligram of tissue; range, 255–27,547 per milligram of tissue; Fig. 2f). Dermal cell infiltration of varying intensity was evident in all cases, and immunostaining for *Leishmania* Oligopeptidase B (OPB) and DAPI staining confirmed parasites in all five volunteers (Fig. 2g–j and Extended Data Fig. 1). In some sections, discrete foci of cellular infiltration were observed, consistent with multiple bites. Responses at these different sites appeared heterogeneous. For example, in LC001, one area of focal infiltration accompanied by epidermal remodeling and with scant parasites was adjacent to an ulcer with extensive underlying parasitism (Fig. 2g,h). By our per-protocol definition (Methods), we calculated the take rate for this cohort as 83% (95% confidence interval (CI): 0.44, 0.97), rising to 100% (95% CI: 0.57, 1) for participants with at least one confirmed bite.

For the second cohort (*n* = 8), we made two procedural changes intended to minimize lesion size and/or subsequent scarring. We explored reducing the biting aperture to 3 mm (LC016), 4 mm (LC011, LC021, LC023 and LC026) or 5 mm (LC020, LC025 and LC027). Although this had no significant effect on the number of bites received (Fig. 2b) or the number of fed flies (Fig. 2c), there was a trend toward a reduction in both indices of transmission compared to cohort 1. We also performed lesion biopsy earlier (median, 18 d), using a 6–8-mm punch biopsy. Two of eight volunteers (25%; LC023 and LC027) were deemed bite failures. LC027 developed a minor localized reaction near the site of sand fly exposure and was biopsied at day 19 for further investigation. Parasite qPCR and IHC were negative; no histological abnormalities were observed; and the participant remained lesion free (Extended Data Fig. 1). Of the remaining six volunteers with confirmed bites, LC021 had a small palpable lesion (maximum diameter, 3.3 mm^2^) that spontaneously resolved by day 42, and no biopsy or qPCR was performed. LC011, LC016, LC020, LC025 and LC026 developed a clinically compatible lesion (median area at days 14–19 of 9.5 mm^2^; range, 0–50.1 mm^2^; Fig. 2e). Early lesion areas were more variable than in cohort 1 but not significantly different (*P* = 0.82, Mann–Whitney test). Parasite load determined by qPCR or IHC was highly variable (Fig. 2f,i). LC020 was negative by qPCR and IHC, and histology lacked focal dermal infiltration and, therefore, did not meet our per-protocol lesion definition, despite being clinically compatible. For LC026, qPCR was positive with a pronounced dermal infiltration, albeit parasites were not observed by IHC. LC016 was qPCR negative but IHC positive with typical CL histology. We estimated take rate for cohort 2 as 50% (95% CI: 0.22, 0.78) or 67% (95% CI: 0.3, 0.90) for those with confirmed bite. Across both cohorts, we determined an overall take rate of 64% (95% CI: 0.39, 0.84) for all participants (9/14) or 82% (95% CI: 0.52, 0.95) for those with confirmed bite (9/11).

### Recurrence after therapeutic biopsy

**Fig. 3:**
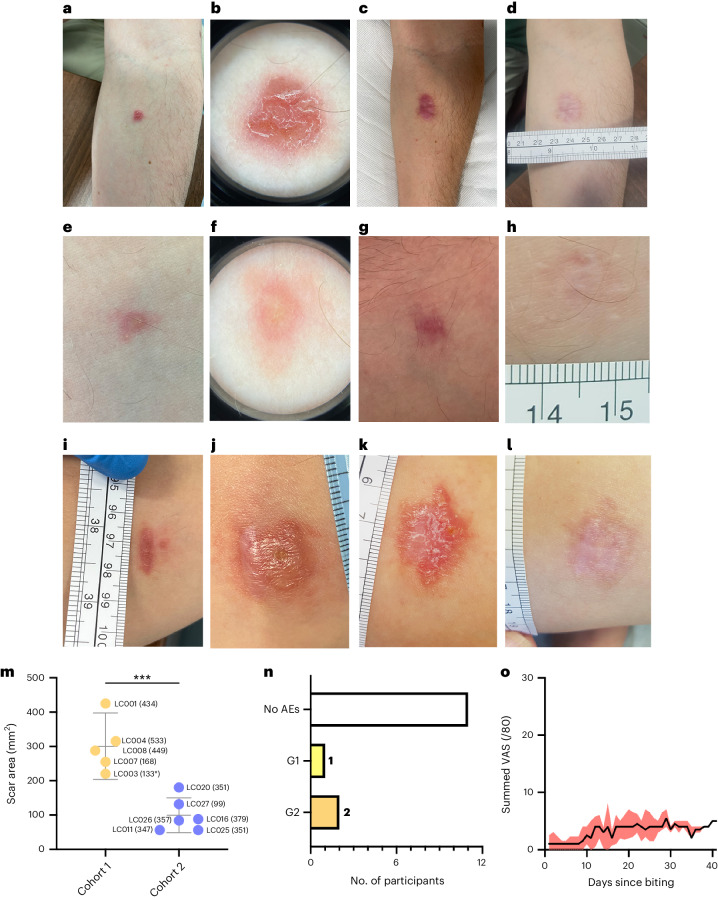
Clinical features of the LEISH_Challenge study. **a**–**d**, Participant LC008 images taken 28 d post-bite (p.b.) and before excision biopsy (**a**,**b**) and 223 d (**c**) and 476 d (**d**) p.b. **e**–**h**, Participant LC011 images taken 42 d p.b. and before 4-mm punch biopsy (**a**,**b**) and 140 d (**c**) and 350 d (**d**) p.b. Dermoscopy images are shown in **b** and **f**. **i**–**l**, Participant LC001 had a primary lesion (excised 37 d p.b), followed by a secondary lesion (4-mm punch biopsy and cryotherapy at 255 d p.b.). Images showing secondary lesion adjacent to healing primary lesion at 161 d (**a**) 251 d (**b**), 309 d (**c**) and 470 d (**d**) p.b. **m**, Quantitation of scar area at final measured follow-up. Data are presented as individual data points (labeled by participant ID and time in days from initial biopsy) with mean and 95% CI. *n* = 5 (cohort 1) and *n* = 6 (cohort 2). ***P = 0.0005 (two-sided Student’s *t*-test). Further details are provided in Supplementary Table 1. **n**, Number of adverse events by grade (G) assigned as definitely, probably or possibly related to the study. Further details are provided in Supplementary Table 1. **o**, Summed participant-recorded VAS across eight parameters (itch, pain, erythema, swelling, malaise, myalgia, fever and nausea). Maximum score available = 80. Data are shown as median and interquartile range (IQR) for all participants. Individual participant scores and summed scores for each parameter are provided in Extended Data Fig. 2. AE, adverse event.

Of the 10 participants biopsied, seven (70%) required no further treatment and remain lesion free (Fig. 3a–h). In three cases (LC001, LC004 and LC016), a lesion subsequently developed at the biopsy site 4–8 months after biopsy (Fig. 3i–l). A second biopsy (punch) was performed for parasitological confirmation (30,800, 184 and 1,658 parasites per milligram of tissue in biopsies taken at 255 d, 282 d and 126 d after bite, respectively). Cryotherapy was initiated using a standard delivery device (0.75-mm nozzle; repeated 10-s freeze–thaw cycle) as per protocol with all three volunteers receiving three treatments spaced over 6–8 weeks. All responded well with apparent healing of their CL lesion. LC004 had a second recurrence 14 months after the original biopsy. This resolved with an additional cycle of cryotherapy.

We considered potential factors influencing recurrence. For LC001, further evaluation of dermoscopy images suggested that one bite was beyond the perimeter of the original biopsy and may have been a slow-to-evolve primary lesion rather than a recurrence (Fig. 3i–l). Participant LC004 confirmed a trauma at the biopsy site before recurrence. This participant remained concerned about the residual hypertrophic scarring after cryotherapy and Adcortyl (0.25 ml of 10 mg ml^−1^) was administered to the scar tissue. A new lesion developed 2 months later, questioning whether the intralesional steroid was a contributory factor. For LC016, no precipitating factors were identified.

### Scarring in CHIM participants

Scarring was evident in all biopsied participants (Supplementary Table 1). Seven (LC007, LC008, LC011, LC016, LC020, LC025 and LC026) had mild atrophic scarring. Two (LC021 and LC027) who developed atypical lesions without parasitological confirmation of CL developed mild atrophic scarring. One (LC003) had moderate scarring. Two (LC001 and LC004) who developed a second CL lesion had more moderate to severe scarring, resulting from the combined effects of CL, the surgical procedure(s) and cryotherapy. LC016 had mild atrophic scarring, perhaps reflecting earlier biopsy, smaller lesion size and use of punch versus elliptical excision biopsy. Two (LC003 and LC004) had a post-biopsy wound infection that may have contributed to scarring. Measurement of the scar area at final follow-up confirmed reduced scarring in cohort 2 (punch biopsy) compared to cohort 1 (excision biopsy) (Fig. 3m). Scar area was similar between females and males across both cohorts (females: median, 200 mm^2^; range, 56–425 mm^2^; males: median, 88 mm^2^; range, 56–315 mm^2^).

### Safety and adverse events

**Extended Data Fig. 2:**
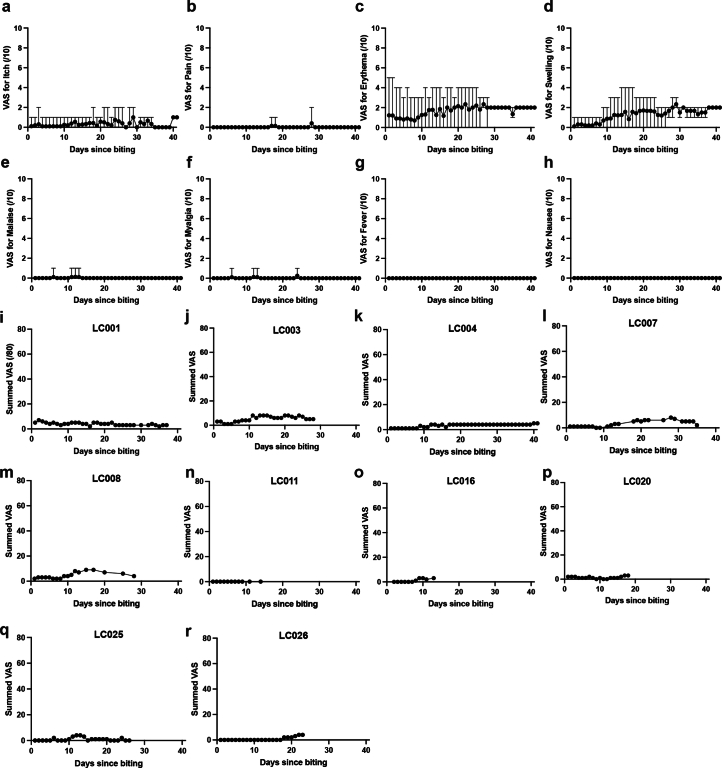
Visual Analogue Scores by feature and participant. **a-h**. VAS scores out of 10 for itch, pain, erythema, swelling, malaise, myalgia, fever and nausea. Data shown as mean and range for all participants with positive bite (n=11). **i-r**. Summed VAS scores for each participant. Data shown as total score out of 80.

No grade 3 or serious adverse events were recorded. No adverse events were reported during the biting phase of the study. One participant had a grade 1 adverse event (exudate from scar), and two had a grade 2 adverse event (wound site infections) (Fig. 3n and Supplementary Table 1). Wound infections were associated with itch and scratching but showed no evidence of cellulitis. For all volunteers, full blood count, liver function tests, urea and electrolytes and C-reactive protein were taken at baseline and follow-up, with no significant differences noted (Supplementary Table 2). A minimal inflammatory response was noted, and any changes observed in the above parameters remained within normal range and were deemed not clinically relevant. All volunteers remained seronegative (rK39) throughout follow-up. Lymphadenopathy (epitrochlear and axillary lymph nodes) was absent in all participants. As previously described^26^, additional safety outcomes were collated using an electronic participant-submitted visual analogue score (VAS) diary card, recording on a 1–10 scale subjective perceptions of itch, pain, erythema, swelling, malaise, myalgia, fever and nausea (Fig. 3o and Extended Data Fig. 2). Summed VASs per participant were not dissimilar to those after the bite of uninfected sand flies^26^. Participants also completed validated quality-of-life questionnaires. The Dermatology Life Quality Index (DLQI) measured the impact of skin changes, and the Generalized Anxiety Disorder 7 (GAD-7) score measured mood disturbances. The mean changes in score for bitten participants were 1.92 ± 2.54 (for DLQI, 30-point scale) and −0.17 ± 1.94 (for GAD-7, 21-point scale). These values were below the minimal clinically important difference (MCID), indicating that this CHIM was generally well tolerated^29,30^.

### Immune landscape of CL lesions

**Fig. 4:**
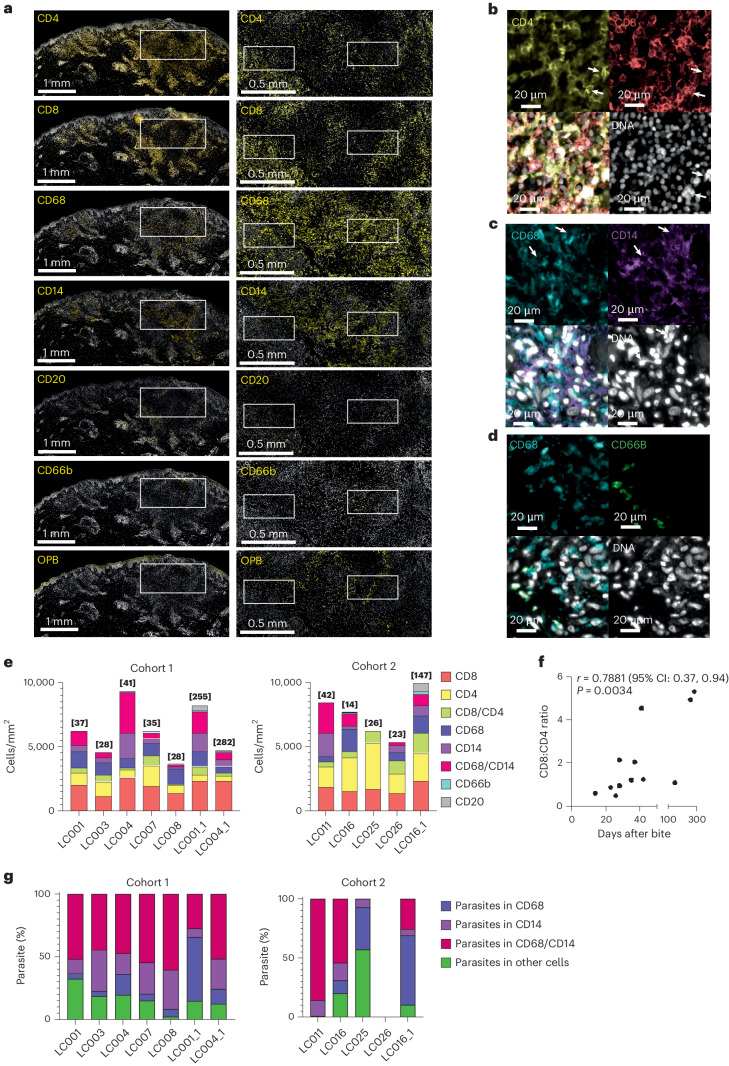
Inflammatory response after *L. major* challenge. **a**, Immuno-histological detection (yellow) of lesion expression of CD4, CD8, CD68, CD14, CD20, CD66b and parasites (OPB), shown for a single participant (LC001). Sections were counterstained for nuclei (YOYO1, white). Scale bars, 1 mm (left images) and 0.5 mm (white box; right images). Higher magnification images for white boxes shown in the right panel are provided in Extended Data Fig. 1, and pairwise staining combinations on serial sections are shown in **b**–**d**. **b**, Representative images of CD4 (yellow), CD8 (red), DNA (white) and merged image. CD4^+^CD8^+^ cells are indicated by arrowheads. **c**, Representative images of CD68 (blue), CD14 (purple), DNA (white) and merged image. Infected CD14^+^CD68^+^ cells are indicated by arrowheads. **d**, Representative images of CD68 (blue) and CD66b (green), DNA (white) and merged image. Uninfected CD66b^+^ cells are seen adjacent to heavily parasitized CD68^+^ cells. **e**, Quantitation of cellular infiltrate across all participants based on IHC. Second biopsies are denoted by participant number followed by _1. Data are shown in stacked bar format with time of biopsy (days post-bite) shown in parentheses above the bar. Data were derived from whole sections. **f**, Correlation between time after biopsy and CD8:CD4 ratio. Data were analyzed using Spearmanʼs two-tailed test. **g**, Proportion of total parasites found in CD14^+^, CD68^+^, CD14^+^CD68^+^ or other cell types. Total number of parasites counted ranged from 327 to 38,693, except for LC025 where only 14 parasites were detected. Data are shown in stacked bar format.

**Extended Data Fig. 3:**
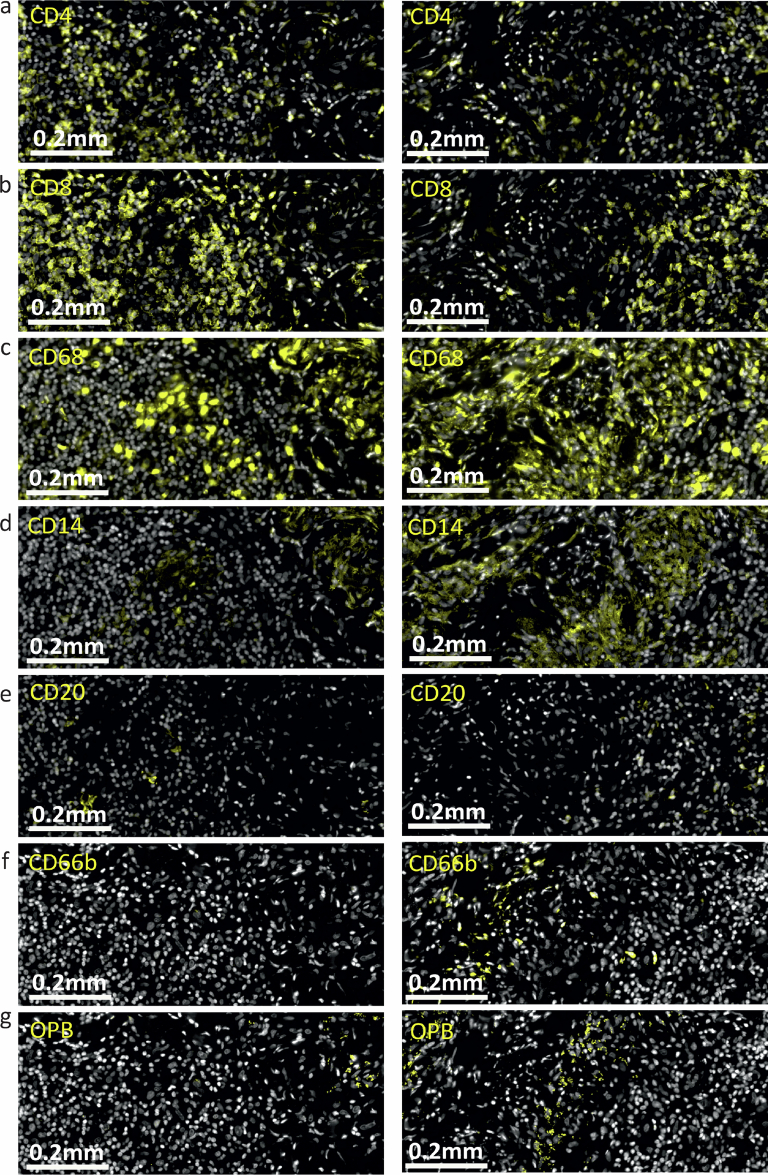
IHC staining for immune cells populations. **a-g**. IHC (yellow) for CD4 (**a**), CD8 (**b**), CD68 (**c**), CD14 (**d**), CD20 (**e**), CD66b (**f**) and Leishmania OPB (**g**). Left and right images correspond to boxed areas of images shown in Fig. 4a right. Images are for a single participant (LC001) and pair wise staining was performed on serial sections. Scale bar in all images represents 0.2 mm.

Histologically, lesions showed one or more characteristic features, including a dense lympho-histiocytic infiltration extending from the papillary into the reticular dermis, acanthosis with elongation of rete ridges (for example, LC007 and LC016), patchy hyperkeratosis (for example, LC008) and occasional unorganized granulomas (for example, LC025). Compact organized granulomas with epithelioid cells and/or Langhans giant cells were not seen. Extensive collagen fibers were often observed (Extended Data Fig. 1). In recurrence biopsies, dermal infiltration was enhanced and reached the hypodermis. CD4^+^ and CD8^+^ cells were detected in all biopsies at variable ratios, and overall CD8:CD4 ratio positively correlated with lesion duration (Spearman’s test; Fig. 4a,b,e,f and Extended Data Fig. 3). CD4^+^CD8^+^ cells were observed (Fig. 4b), consistent with other reports^31,32^. CD14^+^ monocytes and CD68^+^CD14^+^ monocyte-derived macrophages^33^ generally outnumbered CD68^+^ dermal macrophages, but this was not consistent across all participants, and there were no significant correlations with lesion duration (Spearman’s test; Fig. 4a,c,e and Extended Data Fig. 3). Parasitism was largely, but not exclusively, confined to CD14^+^, CD68^+^ and CD14^+^CD68^+^ cells, with more variability observed in cohort 2 (Fig. 4g). There was no correlation between proportion of each infected cell type and duration of infection (Spearman’s test). CD66b^+^ neutrophils were infrequent and mostly confined to sites of active ulceration and epidermal breakdown (Fig. 4a,d and Extended Data Fig. 3). Neutrophils were rarely parasitized but were observed near infected CD68^+^ cells (Fig. 4d). CD20^+^ B cells were scarce, sparsely dispersed and more abundant in recurrent lesions (Fig. 4a,e and Extended Data Fig. 3).

**Fig. 5:**
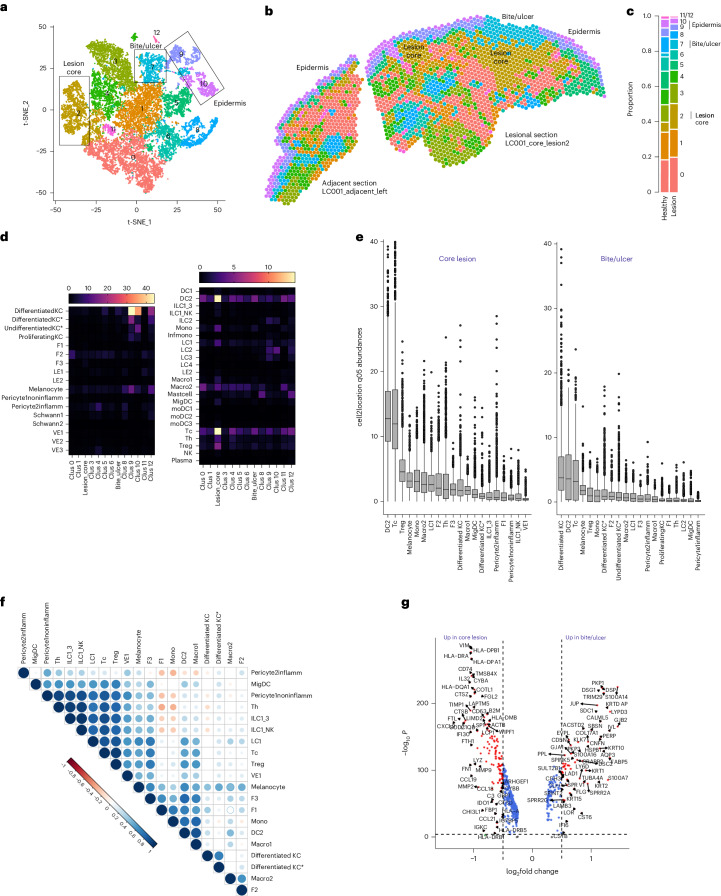
Transcriptomic landscape of early *L. major* lesions. FFPE sections from LC001, LC003 and LC008 were processed for Visium spatial transcriptomics with cell deconvolution performed using cell2location based on skin cell types identified by Reynolds et al.^34^. **a**,**b**, Clustering of spots reveals 13 clusters in UMAP space (**a**) and with discrete spatial locations (**b**). Cluster locations are mapped to lesion and adjacent sections from LC001. Mapping to LC003 and LC008 and additional sections from LC001 are shown in Extended Data Fig. 4. **c**, Proportion of spots attributed to each cluster in healthy and lesion tissue. Data are pooled across all participants/sections. **d**, Heatmap representation of cellular abundances by cluster as determined by cell2location using the Reynolds et al.^34^ reference dataset. Scale represents predicted 5% quantile abundances (q05 = 5% quantile values of the posterior distribution). **e**, Box and whisker plots representing cellular abundances/spot as in **d** for lesion core (cluster 2) and ulcer (cluster 7). Data are shown for the top 20 most abundant cell types. *n* = 20,241 spots derived from four sections from each of three participants. Box bounds show interquartile range (IQR) from the 25th to the 75th percentile; whiskers show the smallest and largest values within 1.5× the IQR from the lower and upper quartiles; and outliers are shown as data points outside the whiskers. **f**, Pairwise Pearson’s correlations are represented as a correlation plot between cell types to infer spatial co-localization. **g**, Volcano plot of differentially expressed genes (log_2_FC > 1.5 and FDR = 0.05) comparing lesion core with ulcer. Statistical analysis was performed using two-sided Wilcoxon rank-sum test with Bonferroni correction for multiple comparisons.

**Extended Data Fig. 4:**
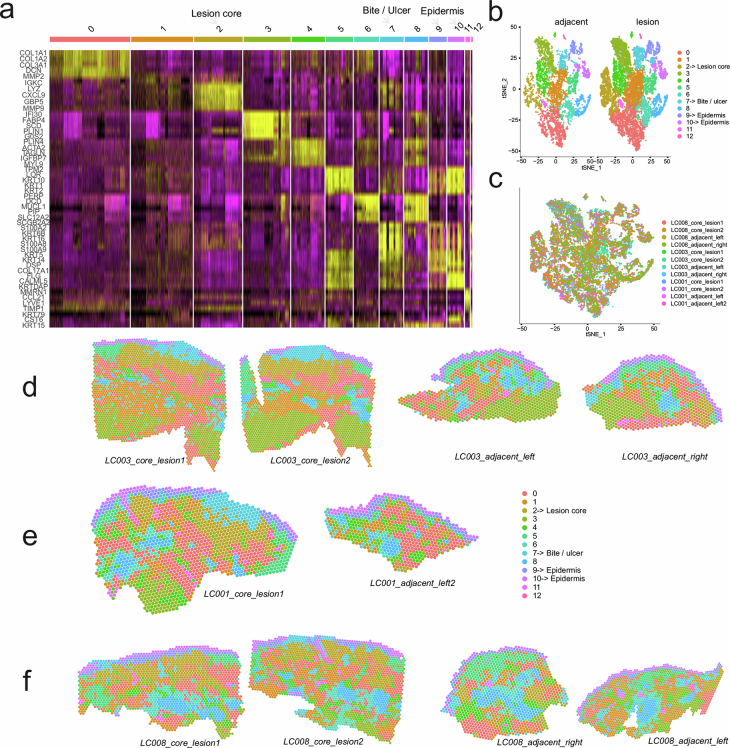
Visium spatial maps of CL lesions. **a**, heat map representation of top 5 genes representing clusters 0–12 in Fig. 5a and 5b. **b**, tSNE representation of clusters by lesion vs adjacent tissue. **c**, tSNE representation showing overlays for all samples studied. **d-f**, spatial mapping of clusters for all sections studied for participant LC003 (**d**), LC001 (**e**) and LC008 (**f**).

**Extended Data Fig. 5:**
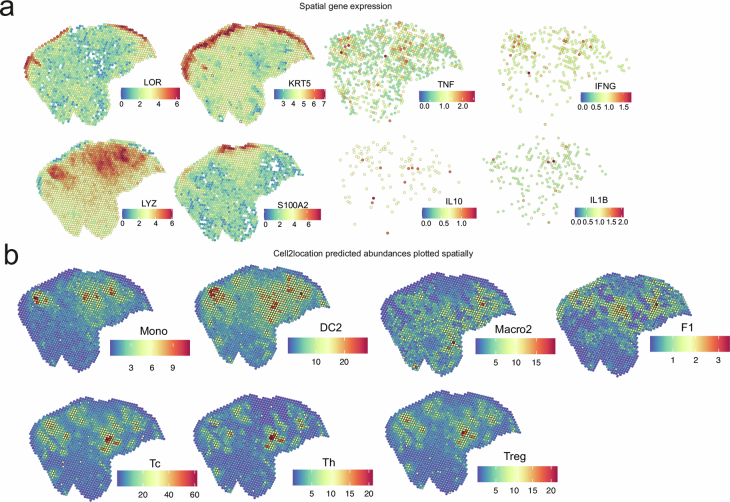
Localisation of select mRNA and predicted cell types. **a**, feature maps showing location of mRNA *LOR*, *KRT5*, *LYZ*, *S100A2*, *TNF*, *IFNG*, *IL10* and *IL1B*. **b**, Feature maps showing cell2location-predicted cells types by abundance for monocytes (Mono), myeloid dendritic cells 2 (DC2), macrophages 2 (Macro2), fibroblasts 1 (F1), cytotoxic T cells (Tc), helper T cells (Th) and regulatory T cells (Treg).

We used Visium spatial transcriptomics (10x Genomics), a skin single-cell RNA sequencing (scRNA-seq) dataset^34^ and the cell2location prediction tool^35^ to interrogate the immune landscape in three participants (LC001, LC003 and LC008) whose biopsies included histologically normal skin. We analyzed 20,241 55-μm-diameter Visium spots from 12 formalin-fixed, paraffin-embedded (FFPE) sections (four per individual) with a median gene content of 2,000 genes per spot. We used t-distributed stochastic neighbor embedding (t-SNE) to visualize spots by Louvain clustering (13 clusters; Fig. 5a and Extended Data Fig. 4) and generated a spatially resolved transcriptomic map of each biopsy (Fig. 5a–c and Extended Data Fig. 4). Cell deconvolution and transcript abundance identified key immune and stromal cell subsets associated with each cluster (Fig. 5d,e, Extended Data Fig. 5 and Supplementary Table 3). Cluster 2 (herein referred to as the lesion ‘core’) had abundant myeloid DC2, MigDC, LC1, Macro1, Macro2 and monocytes as well as Tc, Th and Tregs; was enriched for interferon-inducible genes (*CXCL9* and *GBP5*), *LYZ* and Ig transcripts and effector and regulatory cytokines (*IFNG*, *TNF*, *IL-10* and *IL1B*); and was proportionally overrepresented in lesion compared to healthy tissue (Fig. 5c and Extended Data Fig. 4). Within cluster 2, we also observed spatial co-occurrence of cell types suggestive of further spatial heterogeneity (Fig. 5f). For example, pericytes, MigDC, Th, ILC1_3, ILC1_NK, LC1, Tc, Treg and vascular endothelial cells (VE1) were highly correlated, suggesting common pathways for recruitment, but were strongly anti-correlated with podoplanin-expressing F1 fibroblasts and Mono. Clusters 9 and 10 were enriched for keratinocytes, melanocytes, Langerhans cells (LC1, LC2 and LC3) and ILC2, and, together with clusters 5 and 12 (*CST6*), spatially defined the intact epidermis and epidermal/dermal border. Cluster 7 mapped to the ‘ulcer’ and comprised a mixture of myeloid cells and lymphocytes, with differentiated KC, DC2 and Tregs and a mixed gene signature including keratins, S100 proteins and collagens. Epidermal disruption was evident, with reduced expression of the basal epidermal marker *KRT5* and *LOR* (loricin, a major component of terminally differentiated epidermal cells). Of note, *LOR* mRNA was less abundant in the epidermis overlying the secondary lesion core in LC001, suggesting a less well differentiated epidermis at this site and consistent with a thickening of the stratum spinosum relative to adjacent tissue (Extended Data Fig. 4). Cluster 3, mapped to the deep dermis/hypodermis, contained Macro2 and was notable for genes associated with lipid metabolism (*FABP4*, *SCD*, *PLIN1* and *GOS2*), consistent with the presence of adipose tissue, whereas dermal cluster 4 had smooth muscle cell markers (*MYL9*, *TAGLN* and *ACTA2*), suggestive of proximity to hair follicles. Dermal cluster 0 was rich in mRNAs for extracellular matrix genes (*COL1A1*, *COL1A2*, *COL3A1* and *DCN*) and *MMP2*, with F2 fibroblasts the dominant stromal population. Cluster 1 was notable by an absence of key defining genes, and cluster 11 (*CCL21*, *LYVE1* and various myeloid and T cells) comprised relatively few spots.

Given their leucocyte-rich composition, we compared the lesion core and ulcer in more detail. We identified 134 differentially expressed genes (5% false discovery rate (FDR); >1.5 fold change (FC); Fig. 5g and Supplementary Table 3) between core (80 upregulated) and ulcer (54 upregulated). mRNAs with greater abundance in the core were related to antigen processing and presentation (for example, *HLA-DRA*, *HLA-DPA1* and *CD74*), metalloproteinase activity (*MMP2*, *MMP9* and *TIMP1*), multiple cytokines and chemokines (*CCL5*, *CCL18*, *CCL19* and *CCL21*), the metabolic checkpoint enzyme *IL-4l1* (ref. ^36^) and *IL-32* (associated with IDO1 and PD-L1 expression in CL lesions^37^). Pathway analysis^38^ identified multiple immune-related pathways in the lesion core. Pathways in the ulcer were related to epidermal remodeling (Supplementary Table 3). Discordance between the histological detection of neutrophils (Fig. 3) and enrichment of a neutrophil degranulation pathway in the core may reflect expression of pathway-associated genes by monocytes/macrophages in inflammation (for example, *CTSG*, *MPO*, *CD63* and *MMP9*).

**Fig. 6:**
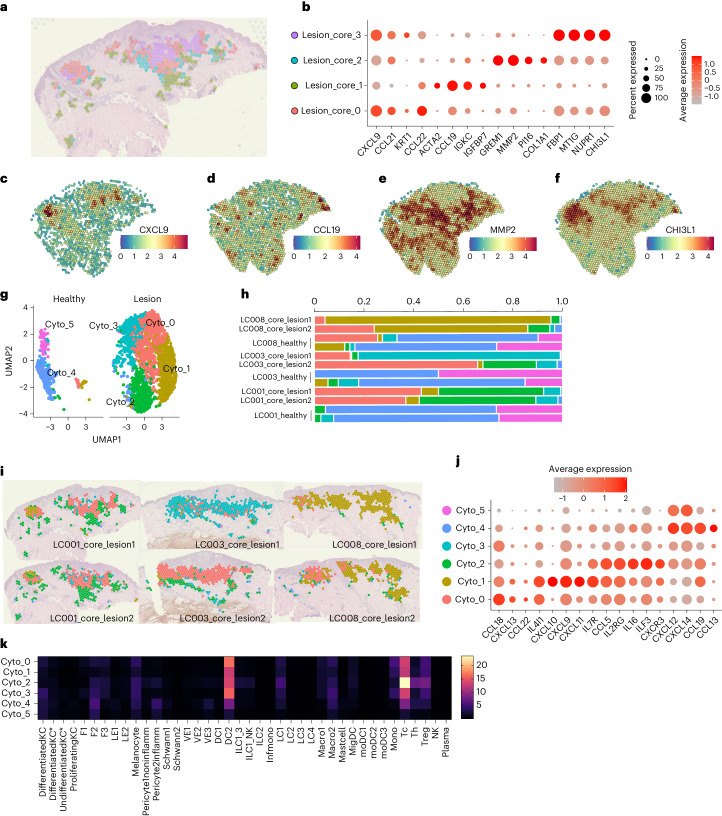
Discrete spatial niches in the lesion core. **a**, Subdivision of the lesion core cluster (Fig. 5a,b) into four subclusters. **b**, Bubble plot showing top genes associated with lesion core subclusters. **c**–**f**, *CXCL9* (**c**), *CCL19* (**d**), *MMP2* (**e**) and *CHI3L1* (**f**) mRNA abundance mapped to LC001 for visualization. **g**,**h**, Re-clustered lesion core (Lesion_core_0/1/2/3 as in **a**,**b**) based on chemokine and cytokine family genes visualized in UMAP space as Cyto_0/1/2/3/4/5 and shown separately for healthy versus lesional tissue (**g**). **h**, Proportion of spots in healthy and lesion tissue shown individually for each section/participant. **i**, Cyto_0/1/2/3 clusters mapped to each participant for visualization. **j**, Bubble plot showing key genes associated with each cytokine/chemokine-based cluster. Scale bar shows average expression. **k**, Predicted cell abundances for Cyto_0–5 clusters, based on data from all sections (*n* = 6) and participants (*n* = 3).

To further characterize the lesion core, we identified spatially distinct subclusters (Fig. 6a,b). Subcluster 0 was interspersed with subcluster 3, with abundant mRNA for antimicrobial and monocyte/T cell chemoattractants (*CCL22* and *CXCL9* (refs. ^39,40^); Fig. 6b,c). mRNAs whose abundance correlated with *CXCL9* comprised a STRING network enriched for Gene Ontology (GO) terms related to IFNγ response, antigen presentation, neutrophil activation and leucocyte adhesion (Supplementary Table 3). Subcluster 1 had abundant mRNA for *CCL19* (Fig. 6b,d) and B cells (*IGKC*) and was located at the core periphery and the deep dermis. Subcluster 2 contained presumptive fibroblasts (*GREM1*, *MMP2* and *PI16*) and was localized to the periphery (Fig. 6b,e). Subcluster 3 was characterized by *CHI3L1* (a chitinase-like protein with broad-ranging activity in inflammation, tissue repair and macrophage polarization^41^; Fig. 6b,f), NUPR1 (strongly expressed by basophils and neutrophils), FBP1 (fructose-1,6-bisphosphatase 1, a marker of human M1 polarization^42^) and MT1G (metallothionein 1G, associated with cancer-associated TREM^hi^ macrophages^43^ and a pleiotropic regulator of myeloid cell function^44^).

Given the importance of chemokines and cytokines in anti-leishmanial immunity, we re-clustered these data (that is, Lesion_core_0–3) based on expression of these molecules alone (Fig. 6g–k). Six clusters (Cyto_0–5) were visualized in uniform manifold approximation and projection (UMAP) space (Fig. 6g). Cyto_0–3 were largely confined to the core, albeit with some variability between participants and across serial sections (Fig. 6h,i). Cyto_0 (*CCL18*) and Cyto_1 (*CXCL9*, *CXCL10* and CXCL11) formed the central region of the core, whereas Cyto_2 (*CCL5*, *CCL19*, *IL-16, IL2RG* and *CXCR3*) was largely confined to its borders (Fig. 6i). Th cells were selectively associated with Cyto_2, in keeping with the function of CCL5 and IL-16 (Fig. 6k), although Tc cells were more abundant. Cyto_3 generally had lower cytokine and chemokine mRNA abundance and was mainly derived from one section (Fig. 6i). Cyto_4 and Cyto_5 (*CXCL12*, *CXCL14* and *CCL13*) mapped mainly to healthy tissue and comprised F2 fibroblasts and various myeloid and lymphocyte populations in low abundance (Fig. 6k). Hence, the lesion core, whether subclustered in an unbiased manner (Fig. 6a,b) or using only cytokine/chemokine genes (Fig. 6g–k), displays clear and hitherto unrecognized functional compartmentalization and cellular heterogeneity.

## Discussion

We report clinical, parasitological and preliminary immunological data from the first CHIM of sand fly-transmitted CL caused by *L. major*. We demonstrate that this model has a take rate similar to that of other CHIMs and is safe and well tolerated by participants, suggesting suitability for evaluating vaccines, pre-exposure or post-exposure therapies and mechanism of immunopathology in humans.

A previous human infection study using needle challenge with *L. major* allowed lesion progression to self-cure, with most participants developing ulcerated lesions by 60 d after inoculation^22^. In contrast, our study was designed to estimate take rate for a cGMP-produced *L. major* strain transmitted by laboratory-reared *P. duboscqi*. Furthermore, we used therapeutic biopsy^45^ to excise the lesion early in development. The latter approach was guided by a public involvement (PI) exercise^27^ and a desire to avoid extensive lesion development. Using the biopsies to increase understanding of the disease was also recognised by our PI group as a desirable outcome^27^. Results from the first cohort estimated a take rate of 83% for all participants (100% for those receiving a bite). Recruitment was extended to increase confidence in this estimate and to incorporate changes aimed at minimizing lesion size and scarring. These contributed to reduced scaring, but the smaller lesions and earlier timepoints of biopsy reduced apparent take rate. Nevertheless, our overall estimate of 64% aligns with other CHIMs and avoids an overwhelming force of infection^46^. If higher take rates were desired (for example, for discovery research or drug evaluation), this might be achievable by increasing the number of infected sand flies or extending exposure time.

Target product profiles for CL vaccines propose an efficacy of 70%^8^. Based on a dichotomous endpoint (lesion versus no lesion), which is perhaps the gold standard for an effective vaccine, and our estimated take rate, the current protocol could provide vaccine efficacy data with approximately 50 participants. Coupled with the rapidity of lesion development, likely facilitated by sand fly transmission, this would be highly cost-effective compared to field trials in endemic countries. Relaxing the endpoint definition, for example by excluding parasitological confirmation, would increase take rate and reduce sample size further. Adopting continuous measures of vaccine efficacy would have consequences for sample size, cost and burden on participants. For example, lesion parasite load was highly variable, reflecting (1) the early timepoints studied, (2) the sampling method, (3) the nature of vector transmission^47,48^ and (4) inter-individual variation in immune response. qPCR of sequential microbiopsies^49^ might mitigate against variation in parasite load but could impact on lesion progression. Similarly, assessment of vaccine-induced reduction in CL scar would require omission of the therapeutic biopsy and participant consent to allow CL to take its full course.

CL results in scarring, and some degree of scarring was evident in all participants who developed a lesion, underwent biopsy and/or received cryotherapy. Reducing the area of exposure and early punch biopsy resulted in better cosmesis, but the study was not designed to evaluate this formally. Conversely, wound infection, later excision biopsy and cryotherapy all appeared to contribute to scarring. The frequency of wound infection was within acceptable limits but could be further mitigated using antimicrobial washes (for example, Dermol 500) and antihistamines. All suspected wound infections responded quickly to treatment. Regarding mechanisms of scar formation, preliminary analysis identified epidermal remodeling and abundant mRNA for mediators of fibrosis (*MMP9*) previously associated with clinical outcome in South American CL and VL^50,51^. However, in the absence of a control arm, it is difficult to formally distinguish between scarring attributable to CL and that resulting from biopsy. Such a study could be considered in the future to generate new insights into CL scar formation.

This study has additional limitations. In participants with recurrence after therapeutic biopsy, each responded well to cryotherapy. In one case, steroid administration may have been a precipitating factor for a further recurrence, suggesting that intralesional steroid injection should be contraindicated. Although the possibility of future recurrence cannot be completely excluded, the risk appears low. Relapse of *L. major* due to HIV-associated or elective immunosuppression has not been reported, and, unlike in mice, *L. major* does not appear to persist in the scars of patients with CL^52^. All participants in the study were White, and future CHIM studies should consider race, ethnicity and environment. Constraints to implementing CHIM studies in lower-income and middle-income settings^53^ apply equally to this CHIM, and additional considerations related to vector diversity and disease heterogeneity have been discussed elsewhere^54,55^. Finally, we performed biopsy over a narrow time window, consistent with the study objectives, but timings could be readily altered to accommodate different objectives, for example to study innate immunity or more advanced immunopathology.

*L. major* infection in mice helped to establish the Th1/Th2 paradigm of cellular immunity^56^ and to understand vector contributions to pathogenesis^23,57^ and the contribution of myeloid cells to CL chronicity^58^. Studies on human immunity to *L. major* are, however, less comprehensive. Cure is associated with Th1-mediated IFNγ responses that promote self-healing^59,60^. Immunohistochemical studies described altered adhesion molecule and major histocompatibility complex (MHC) expression associated with infiltration by CD4^+^ and CD8^+^ T cells^61^. Other studies^62,63^ reported detection of mRNA for *IFNG*, *TNF* and *IL6* and less frequent detection of *IL4* and suggested that IL-10 might support an immunosuppressive milieu^58,62^. However, these and similar studies provide an incomplete picture of the immune landscape and are limited to patients with well-established lesions. In contrast, our biopsies provided a unique opportunity to examine early lesion progression after natural infection. Our initial analyses highlight how immune responses differ between the lesion core and ulcer, indicative of the independence of anti-parasitic and wound healing responses. We observed an unexpectedly high frequency of CD8^+^ T cells, particularly in recurrent or late lesions, and the predominant parasitism of macrophages and monocyte-derived macrophages, and we predicted DC2 as the dominant myeloid cell type in the lesion core. We identified diverse immune niches with selective chemokine/cytokine expression linked to cellular composition and various stages of epidermal remodeling associated with ulceration or underlying inflammation. Collectively, these data provide a blueprint to determine how microenvironment shapes infection over time, to identify correlates of protection and pathology and to inform the development of vaccines, drugs and host-directed therapies though mechanistic understanding of immunity.

In conclusion, we report a safe and effective CHIM based on natural transmission of *L. major*. Notwithstanding disease heterogeneity^1^, epidemiological and experimental evidence supports at least some natural or vaccine-induced cross-species protection^12,64^. Hence, this CHIM will have broad utility for assessing vaccines designed to target many forms of leishmaniasis, including VL. Our analyses also highlight functional compartmentalization of immune responses at the site of infection and provide a resource to comprehensively map the immune landscape in human disease.

## Methods

### Ethics and inclusion statement

The study was approved by the UK Health Research Agency South Central–Hampshire A Research Ethics Committee (IRAS Project ID: 286420; 20/SC/0348) and the Hull York Medical School Ethical Review Committee (approval no. 2073). The study sponsor was the University of York. The study was prospectively registered at ClinicalTrials.gov (identifier: NCT04512742). Participants in this study were both male and female sex. Participants were compensated as defined in the protocol (Extended Data Fig. 3).

### Vectors and parasites

*P. duboscqi* originating in Senegal were maintained in the insectary of the Department of Parasitology, Charles University in Prague, under standard conditions (26 °C on 50% sucrose solution, humidity in the insectary 60–70% and 14-h light/10-h dark photoperiod). Colonies were negative by PCR for sand fly-associated phleboviruses (including sandfly fever Sicilian virus group, Massilia virus and Toscana virus) and flaviviruses (targeting a conserved region of the NS5 gene). As required, batches of approximately 200 sand flies were shipped at 3–5 d of adult development to the University of York in a humidity-controlled and temperature-controlled sealed unit.

After arrival, the sand flies were maintained on a sugar solution for 24 h and subsequently starved to encourage later blood feeding. Twelve to fifteen days before a scheduled biting day, sand flies were infected using a membrane feeder (Hemotek) containing rabbit blood mixed with 10^6^ promastigotes per milliliter of a recently described strain of *L. major*, isolated in Israel and manufactured to cGMP (MHOM/IL/2019/MRC-02 (ref. ^25^)). Three to five days before a scheduled biting day, a sample of engorged sand flies was dissected to ensure infection rates above 90% by standard methods. On the day of the biting study, a sand fly biting chamber (Precision Plastics) was loaded with five female sand flies and placed on the participant’s arm for 30 min. Biting failure was defined by absence of (1) participant-reported biting sensation during and immediately after biting; (2) sand fly biting activity as noted by clinical investigators (including inspection of video and photography during biting); (3) bite-compatible lesions by dermoscopy or photography immediately after biting; and (4) any macroscopic evidence of blood in sand fly abdomen at end of biting period. No discrimination was made between partially and fully fed flies. Volunteers with suspected biting failure were followed up until day 28 and then replaced in the study (with a final follow-up at 6 months).

### Clinical procedures

All clinical procedures and standard operating procedures are provided in the study protocol (Supplementary Information).

### Histology and qPCR

Biopsies were obtained using either a standard elliptical excision biopsy or a punch biopsy. Immediately after biopsy, the tissue was cut into three pieces (50% for histology, 25% for qPCR and 25% for immunological analysis). Extraction of total DNA was performed using a DNeasy tissue isolation kit (Qiagen) according to the manufacturer’s instruction. Parasite quantification by qPCR was performed in a Bio-Rad iCycler iQ Real-Time PCR System using the SYBR Green detection method (SsoAdvanced Universal SYBR Green Supermix, Bio-Rad). Primers targeting 116-bp-long kinetoplast minicircle DNA sequence (forward primer (13A): 5′-GTGGGGGAGGGGCGTTCT-3′ and reverse primer (13B): 5′-ATTTTACACCAACCCCCAGTT-3′) were used^65^. One microliter of DNA was used per individual reaction. PCR amplifications were performed in triplicates using the following conditions: 3 min at 98 °C, followed by 40 repetitive cycles: 10 s at 98 °C and 25 s at 61 °C. PCR water was used as a negative control. A series of 10-fold dilutions of *L. major* promastigote DNA, ranging from 1 × 10^6^ to 1 × 10 parasites per PCR reaction, was used to prepare a standard curve. Quantitative results were expressed by interpolation with a standard curve. To monitor non-specific products or primer dimers, a melting analysis was performed from 70 °C to 95 °C at the end of each run, with a slope of 0.5 °C/c and 5 s at each temperature.

Samples for FFPE were placed in 4% formaldehyde (Thermo Fisher Scientific, 28908) for 24 h at 4 °C. They were then paraffin embedded in Histosette I tissue processing/embedding cassettes (Simport, M490-5) on a Leica ASP300S Fully Enclosed Tissue Processor (Leica Biosystems) and embedded on a Leica EG1150 H Modular Tissue Embedding Center (Leica Biosystems). Blocks were chilled before sectioning. Next, 7-μm sections were cut on a Leica Wax Microtome and placed into a water bath set to 45 °C for 15 s. Sections were then collected onto Superfrost slides (Thermo Fisher Scientific, J1800AMNZ) and allowed to dry overnight at room temperature. Slides were heat fixed at 60 °C for 2 h in a sterilizing oven (Leader Engineering, GP/30/SS/250/HYD, 08H028). Slides were allowed to cool down and then deparaffinized with Histo-Clear II (SLS, NAT1334) for 5 min. Slides were equilibrated in 95% ethanol for 3 min, 70% ethanol for 3 min and distilled water for 3 min.

### Hematoxylin and eosin

Slides were then stained in Harris Hematoxylin (Thermo Fisher Scientific, 6765001) for 3 min and then rinsed in tepid water for 5 min. Slides were dipped once in 1% acid-alcohol (HCl-EtOH, Sigma-Aldrich, 30721-2.5L-M; Thermo Fisher Scientific, E/0650DF/C17) and then equilibrated in distilled water for 3 min. Slides were then stained with 1% eosin (Sigma-Aldrich, E4382-25G) for 3 min and then dipped in 50% ethanol 10 times. Slides were then equilibrated in 70% ethanol for 3 min, 95% ethanol for 3 min and 100% ethanol for 3 min. Slides were then cleared in Histo-Clear II (SLS, NAT1334) for 9 min. Slides were then mounted with dibutylphthalate polystyrene xylene (DPX, Sigma-Aldrich, 06522-500ML) and coverslipped with 22 ×50-mm coverslips (SLS, MIC3226). Slides were dried overnight before being scanned on an Axioscan Z1 (Zeiss).

### IHC

Slides were subjected to heat-mediated antigen retrieval in 10 mmol L^−1^ sodium citrate buffer (pH 6). Sections were incubated with 1% BSA, 0.1% cold fish gelatin and 0.1% Triton X-100 in PBS for 1 h at room temperature to block non-specific immunoglobulin binding. Sections were stained with the following primary antibodies overnight at 4 °C: mouse anti-human CD3 (1:100, OriGene, UM500048CF); rabbit anti-CD4 (1:50, Abcam, ab133616); mouse anti-CD8 (1:100, BioLegend, 372902); rabbit anti-human CD68 (1:800, Abcam, ab213363); mouse anti-CD14 (1:200, Abcam, ab181470), *Leishmania* OPB (10 µg ml^−1^, provided by Jeremy Mottram, University of York); rabbit IgG isotype control (concentration same as the primary, Abcam, ab172730); and mouse IgG1 isotype control (concentration same as the primary, BioLegend, 401401). Primary antibodies were detected by Alexa Fluor 555- labelled F(ab′)2-goat anti-mouse IgG (H+L) cross-adsorbed secondary antibody (Thermo Fisher Scientific, A21425, 1:2,000); Alexa Fluor 647-labelled donkey anti-sheep IgG (H+L) cross-adsorbed secondary antibody (Thermo Fisher Scientific, A21448, 1:2,000); and CF750-labelled donkey anti-rabbit IgG (H+L) highly cross-adsorbed secondary antibody (Biotium, 20298, 1:2,000). All secondary Abs were incubated for 30 min at room temperature. Subsequently, sections were stained with the following conjugated antibodies: mouse anti-CD20 Alexa Fluor 647 (1:100, Novus, NBP-47840C); mouse anti-CD66b Alexa Fluor 647 (1:50, BioLegend, 392912); mouse IgG1 Alexa Fluor 647 (concentration same as the conjugated primary, BioLegend, 400130); and YOYO-1 (Thermo Fisher Scientific, Y3601) for 1 h at room temperature. Sections were mounted in ProLong Gold antifade mountant (Invitrogen, P36930). Images were acquired using a Zeiss Axioscan Z1 slide scanner. Identical exposure times and threshold settings were used for each channel on all sections of similar experiments. Quantification was performed using StrataQuest analysis software (TissueGnostics).

### Visium whole transcriptome spatial transcriptomics and processing

FFPE sections were cut onto 10x Genomics Visium slides with large sections being split into ‘lesion’ and ‘adjacent’ tissue to fit within Visium fiducial markers. Slides were processed according to the Visium Spatial Gene Expression Reagent Kit for FFPE recommended protocol, version 1 (10x Genomics). In brief, slides were stained with hematoxylin and eosin (H&E), imaged and de-crosslinked. Human probes were added overnight and then extended and released. Libraries were prepared according to the manufacturer’s instructions and sequenced using the NovaSeq 6000 platform. Raw FASTQ files were aligned to the human genome GRCh38 (GENCODE version 32/Ensembl 98) using Space Ranger software (10x Genomics). Associated image files were aligned onto slide-specific fiducials using Loupe browser software (10x Genomics). Tissue regions were manually selected, and a tissue *x*–*y* coordinate JSON file was created. JSON files and image files were provided as input to the Space Ranger count() function to generate counts and align them to spatial spots. Raw counts were normalized and analyzed further.

### Normalization and data integration

Seurat (version 4.3.0) was used to find variable features and to normalize and scale the data using the SCTransform() function, and nCount_Spatial and nFeature_Spatial were used to regress the counts. Next, spatial data for four sections per volunteer (three volunteers: LC001, LC003 and LC008) were integrated into one single Seurat object containing 12 images by first selecting features for integration using SelectIntegrationFeatures(), next identifying anchors using FindIntegrationAnchors() and, then, integrating using IntegrateData(). Finally, the first 15 principal components and a resolution of 0.3 were used to obtain cluster memberships per spot. Additionally, underlying histology and clustree^66^ were used to visualize and choose the resolution of clustering. To exclude borderline areas between ‘lesion’ and ‘adjacent’ tissue, H&E images were used to exclude spots that were underlying morphologically altered or disrupted epithelium, as these likely reflected the edge of the lesion. Analysis of spots underlying morphologically normal epithelium were taken to reflect ‘healthy’ tissue for the purposes of comparative analysis. Differential gene expression was calculated first by using the minimum of the median unique molecular identifier (UMI) of individual objects to reverse individual SCT models as a covariate for sequencing depth using the function PrepSCTFindMarkers(). Next, Wilcoxon rank-sum test was employed to find the features that were differentially expressed using an adjusted (Bonferroni correction) *P* value threshold of 0.05. Differentially expressed gene names were submitted to StringDB (https://string-db.org/). The full STRING network (the edges indicate both functional and physical protein associations) was selected for the analysis. *k*-means clustering was performed within STRING to generate three clusters. Pathway analysis was conducted using g:Profiler^38^.

### Cell type deconvolution of Visium spots

We used Reynolds et al.^34^ as a source of single-cell RNA cells from healthy and inflamed skin to model cell abundance per Visium spot using cell2location^35^. cell2location was used as per its recommended instructions. In brief, 50,000 single-cell transcriptomes (retaining cell type annotation as per Reynolds et al.) were used to model reference cell type gene expression using cell2location’s negative binomial regression for 1,000 epochs. Spatial gene expression was then ascribed to cellular abundances based by training the cell2location model for 30,000 epochs. Hyperparameters N_cells_per_location and detection_alpha were selected as 30 and 20, respectively. Finally, predicted abundances (5% quantile values of the posterior distribution) per Visium spot were imported as metadata onto the Seurat object. Predicted abundances were further analyzed by calculating Pearson’s correlation between cell types to suggest co-localization.

### Quantification and statistical analysis

This was an observational exploratory clinical study and was not powered to detect differences in outcome measures between cohorts or between sex, age or other demographic variables. Sample size was chosen on a pragmatic basis to confidently assess attack rate (lower 95% CI of approximately 60%) with the minimum number of participants. Where quantitative measures were analyzed, data were tested using GraphPad Prism (version 10.0.3) for normality (D’Agostino and Pearson or Shapiro–Wilk tests) or assessed using QQ plots. Where underlying distribution was not known, Spearman’s test was used to calculate monotonic relationships. Pearson’s correlation was used, where indicated, to understand linear relationships between cell type co-abundances in space. Before applying Pearson’s test, variable distribution was assessed against theoretical normal distributions using QQ plots. Transcriptomic data were analyzed using appropriate R packages (see above). No blinding was performed, but all downstream analyses of tissue samples were conducted using automated quantitative pipelines (see above).

### Reporting guidelines

See CONSORT diagram and checklist (Fig. 1 and Extended Data Fig. 1).

### Consent

Written informed consent for publication of pseudo-anonymized details and images was obtained from all participants.

### Reporting summary

Further information on research design is available in the Nature Portfolio Reporting Summary linked to this article.

## Online content

Any methods, additional references, Nature Portfolio reporting summaries, source data, extended data, supplementary information, acknowledgements, peer review information; details of author contributions and competing interests; and statements of data and code availability are available at 10.1038/s41591-024-03146-9.

## Supplementary information

CONSORT checklist, Protocol and Patient information sheets.

Reporting Summary

Supplementary Table 1 Demographics, scarring and adverse events. Tab 1, Demographics. Tab 2, Scarring. Tab 3, Adverse events.

Supplementary Table 2 Blood biochemistry and complete blood count data. Data are shown for all participants at each study visit where blood was drawn.

Supplementary Table 3 Visium deconvolution and pathway analysis. Tab 1, Data from Reynolds et al.^34^ used for cell2location deconvolution. Remaining tabs provide pathway analysis and data underpinning Figs. 5 and 6.

## Data Availability

The additional datasets generated, analyzed and that support the conclusions of this study are available from the authors, with agreement from the study sponsor (University of York). Data access requests should be directed to michael.barber@york.ac.uk. Raw transcriptomic data have been deposited in the Gene Expression Omnibus (GSE263298). Processed spatial transcriptomics data are available at 10.5281/zenodo.10018477. The Reynolds et al.^34^ dataset used as a source for scRNA-seq data from healthy and inflamed skin is available at 10.1126/science.aba6500. Human genome (GRCh38; GENCODE version 32/Ensembl 98) raw FASTQ files are available at https://www.gencodegenes.org/human/release_32.html.

## Code availability

Instructions and code are available at https://github.com/jipsi/chim.
