# Supplementary material for: Safety and reactogenicity of a controlled human infection model of sand fly-transmitted cutaneous leishmaniasis
Source: Nat Med. 2024 Aug 2;30(11):3150–62. doi: 10.1038/s41591-024-03146-9 (PMC11564116; doi:10.1038/s41591-024-03146-9)
Supplement: Supplementary file 2 — Reporting Summary [file 41591_2024_3146_MOESM2_ESM.pdf]

nature portfolio

Corresponding author(s): Paul Kaye

Last updated by author(s): Jun 15, 2024

Reporting Summary

Nature Portfolio wishes to improve the reproducibility of the work that we publish. This form provides structure for consistency and transparency in reporting. For further information on Nature Portfolio policies, see our Editorial Policies and the Editorial Policy Checklist.

Statistics

For all statistical analyses, confirm that the following items are present in the figure legend, table legend, main text, or Methods section.

n/a	Confirmed	
☐	☒	The exact sample size (*n*) for each experimental group/condition, given as a discrete number and unit of measurement
☐	☒	A statement on whether measurements were taken from distinct samples or whether the same sample was measured repeatedly
☐	☒	The statistical test(s) used AND whether they are one- or two-sided<br>*Only common tests should be described solely by name; describe more complex techniques in the Methods section.*
☐	☒	A description of all covariates tested
☐	☒	A description of any assumptions or corrections, such as tests of normality and adjustment for multiple comparisons
☐	☒	A full description of the statistical parameters including central tendency (e.g. means) or other basic estimates (e.g. regression coefficient) AND variation (e.g. standard deviation) or associated estimates of uncertainty (e.g. confidence intervals)
☒	☐	For null hypothesis testing, the test statistic (e.g. *F*, *t*, *r*) with confidence intervals, effect sizes, degrees of freedom and *P* value noted<br>*Give P values as exact values whenever suitable.*
☒	☐	For Bayesian analysis, information on the choice of priors and Markov chain Monte Carlo settings
☒	☐	For hierarchical and complex designs, identification of the appropriate level for tests and full reporting of outcomes
☒	☐	Estimates of effect sizes (e.g. Cohen's *d*, Pearson's *r*), indicating how they were calculated

Our web collection on statistics for biologists contains articles on many of the points above.

Software and code

Policy information about availability of computer code

Data collection	Detailed information provided here https://github.com/jipsi/chim and also provided as Parkash et al_NMED_Code_Instructions.pdf
Data analysis	Detailed information provided here https://github.com/jipsi/chim and also provided as Parkash et al_NMED_Code_Instructions.pdf. Software used for data analysis includes Space Ranger (10X Genomics), Loupe Browser (10X Genomics) , StrataQuest (TissueGnostics), Seurat v4.3.0, StringDB (https://string-db.org/), gprofiler (https://biit.cs.ut.ee/gprofiler/gost), cell@location (https://www.sanger.ac.uk/tool/cell2location/), Prism 10.0.3 (GraphPad),

For manuscripts utilizing custom algorithms or software that are central to the research but not yet described in published literature, software must be made available to editors and reviewers. We strongly encourage code deposition in a community repository (e.g. GitHub). See the Nature Portfolio guidelines for submitting code & software for further information.

March 2021

Data

Policy information about availability of data

All manuscripts must include a data availability statement. This statement should provide the following information, where applicable:

- Accession codes, unique identifiers, or web links for publicly available datasets
- A description of any restrictions on data availability
- For clinical datasets or third party data, please ensure that the statement adheres to our policy

The additional datasets generated, analysed, and supporting the conclusions of this study are available from the authors, with agreement from the study sponsor (University of York). Data access requests should be directed to michael.barber@york.ac.uk. Raw transcriptomic data has been deposited in GEO (GSE263298) available from: https://www.ncbi.nlm.nih.gov/geo/query/acc.cgi?acc=GSE263298. Processed spatial transcriptomics data is available at doi:10.5281/zenodo.10018477
The Reynolds et al (Ref 34) used as a source for single-cell RNA-seq data from healthy and inflamed skin is available at doi: 10.1126/science.aba6500.
Human genome (GRCh38; GENCODE v32/Ensembl 98) raw fastq files are available at https://www.gencodegenes.org/human/release_32.html.

Human research participants

Policy information about studies involving human research participants and Sex and Gender in Research.

Reporting on sex and gender	Study was not designed to specifically address sex as a variable but equal numbers of males and females were recruited (Extended Data 2). No analysis based on sex was performed.
Population characteristics	Study recruited healthy volunteers aged 18-50 . Full inclusion and exclusion criteria are provided in Extended Data 3 (protocol).
Recruitment	Participants responded to general advertisement of the study vi.a poster, web pages and local press
Ethics oversight	UK Health Research Agency and Hull York Medical School Ethical Review Committee

Note that full information on the approval of the study protocol must also be provided in the manuscript.

Field-specific reporting

Please select the one below that is the best fit for your research. If you are not sure, read the appropriate sections before making your selection.

☒ Life sciences ☐ Behavioural & social sciences ☐ Ecological, evolutionary & environmental sciences

For a reference copy of the document with all sections, see nature.com/documents/nr-reporting-summary-flat.pdf

Life sciences study design

All studies must disclose on these points even when the disclosure is negative.

Sample size	Sample size was determined pragmatically to minimize participant numbers but provide reasonable confidence that the model was reproducible and fit for purpose (lower bound CI of attack rate of approximately 60%)
Data exclusions	There were no data exclusions
Replication	This was a first evaluation of the feasibility, safety and effectiveness of a new human challenge model. No formal replication was performed but the study was conducted with two independent cohorts showing similar outcomes.
Randomization	No randomisation was performed as the study was open label and observational and no formal analysis was required between cohorts
Blinding	No blinding was performed as this was an open label observational study, with no formal comparisons between groups.. All down stream analysis of tissue samples were conducted using automated pipelines to avoid bias.

Reporting for specific materials, systems and methods

We require information from authors about some types of materials, experimental systems and methods used in many studies. Here, indicate whether each material, system or method listed is relevant to your study. If you are not sure if a list item applies to your research, read the appropriate section before selecting a response.

Materials & experimental systems

n/a	Involved in the study
☐	☒ Antibodies
☒	☐ Eukaryotic cell lines
☒	☐ Palaeontology and archaeology
☒	☐ Animals and other organisms
☐	☒ Clinical data
☒	☐ Dual use research of concern

Methods

n/a	Involved in the study
☒	☐ ChIP-seq
☒	☐ Flow cytometry
☒	☐ MRI-based neuroimaging

Antibodies

Antibodies used	Primary antibodies: mouse anti-human CD3 (1:100, OriGene, UM500048CF), rabbit anti-CD4 (1:50, Abcam USA, Ab133616), mouse anti-CD8 (1:100, Biolegend 372902), rabbit anti-human CD68 (1:800, Abcam USA, ab213363), mouse anti-CD14 (1:200, Abcam USA, ab181470), Leishmania Oligopeptidase B (10µg/ml, provided by Jeremy Mottram, University of York, UK) , rabbit IgG isotype control (concentration same as the primary, Abcam USA, ab172730), and mouse IgG1 isotype control (concentration same as the primary ,BioLegend USA, 401401), mouse anti-CD20 Alexa Fluor 647 (1:100, Novus, NBP-47840C), mouse anti-CD66b Alexa Fluor 647 (1:50, Biolegend, 392912), Mouse IgG1 Alexa Fluor 647 (concentration same as the conjugated primary, Biolegend 400130). Secondary antibodies (at 1:2000): F(ab')2-goat anti-mouse IgG (H+L) cross-adsorbed secondary antibody, Alexa Fluor 555 (Thermo Fisher Scientific, USA, A21425), donkey anti-sheep IgG (H+L) cross-adsorbed secondary antibody, Alexa Fluor 647 (Thermo Fisher Scientific, USA, A21448) and donkey anti-rabbit IgG (H+L) highly cross-adsorbed secondary antibody, CF750 (Biotium, 20298).
Validation	Primary antibodies were used as specified by the manufacturer . pecificity demonstrated for IHC , with controls as indicated in product data sheets.

Clinical data

Policy information about clinical studies

All manuscripts should comply with the ICMJE guidelines for publication of clinical research and a completed CONSORT checklist must be included with all submissions.

Clinical trial registration	ClinicalTrials.gov Identifier: NCT04512742
Study protocol	Supplementary Information pdf
Data collection	Site: University of York Translational Research Facility, York, UK. First and last participants were exposed to infected sand flies on 24th January and 12th August 2022 respectively. Data was collected over a 12 month follow up period.
Outcomes	Primary and secondary outcomes pre-defined in Protocol to assess CL lesion development and safety (primary objectives) and rate of lesion development and immunological analysis of lesion site (secondary objectives). Defined in Supplementary Information pdf.
